# A Panel of Cancer Testis Antigens and Clinical Risk Factors to Predict Metastasis in Colorectal Cancer

**DOI:** 10.1155/2014/272683

**Published:** 2014-03-09

**Authors:** Ramyar Molania, Frouzandeh Mahjoubi, Rezvan Mirzaei, Saeed-Reza Khatami, Bahar Mahjoubi

**Affiliations:** ^1^Division of Medical Biotechnology, National Institute of Genetic Engineering and Biotechnology (NIGEB), Pajoohesh Boulevard, Tehran-Karaj Highway, 17th Km, P.O. Box 14965-161, Tehran, Iran; ^2^Department of Genetics, Faculty of Science, Shahid Chamran University, Ahwaz, Iran; ^3^Hazrat Rasool Hospital, Tehran University of Medical Sciences and Health Care Services, Tehran, Iran

## Abstract

Colorectal cancer (CRC) is the third common carcinoma with a high rate of mortality worldwide and several studies have investigated some molecular and clinicopathological markers for diagnosis and prognosis of its malignant phenotypes. The aim of this study is to evaluate expression frequency of *PAGE4*, *SCP-1*, and *SPANXA/D* cancer testis antigen (CTA) genes as well as some clinical risk markers to predict liver metastasis of colorectal cancer patients. The expression frequency of *PAGE4*, *SCP-1*, and *SPANXA/D* cancer/testis antigen (CTA) genes was obtained using reverse transcription polymerase chain reaction (RT-PCR) assay in 90 colorectal tumor samples including both negative and positive liver metastasis tumors. Statistical analysis was performed to assess the association of three studied genes and clinical risk factors with CRC liver metastasis. The frequency of *PAGE4* and *SCP-1* genes expression was significantly higher in the primary tumours with liver metastasis when statistically compared with primary tumors with no liver metastasis (*P* < 0.05). Among all clinical risk factors studied, the lymph node metastasis and the depth of invasion were statistically correlated with liver metastasis of CRC patients. In addition, using multiple logistic regression, we constructed a model based on *PAGE4* and lymph node metastasis to predict liver metastasis of CRC.

## 1. Introduction

Colorectal cancer (CRC) is the third common cancer and the fourth cause of mortality throughout the world [1]. Accumulating evidence shows that CRC can metastasise to many organs and CRC patients die mainly from metastatic disease. Liver is the preferential target of the CRC metastasis [2]. Nearly 10–25% of CRC patients on diagnosis have liver metastases. However, 20–50% of CRC patients with no detectable metastasis at the time of resection of the primary tumor will develop liver metastases later on [3–5]. Liver resection in CRC patients with liver metastasis remains the best treatment option and it is associated with a survival rate and a 20–25% of long-term survivors [6]. However, without treatment, the median overall survival is approximately 9 months after the recognition of liver metastases [6]. To boost the survival rate of CRC patients, selection of patients at high risk for liver metastasis is crucial.

Two of the most important conventional pathological risk factors for diagnosis of colorectal liver metastasis (CLM) are lymph node metastasis and lymphatic invasion [7]. Molecular studies have shown that there are some useful markers for predicting CLM. Several tumor-associated antigens (TAAs) have been identified in colorectal cancer [8–10]. These TTAs may also be presented in normal cells that show lack of tumor specificity. Another category including tumor-specific antigens (TASs) is unique to the tumor and is not produced by any type of normal cells. The most important subgroup of TSA is cancer testis antigens (CTAs) expressed in the normal testis tissue and some types of cancers. Due to their restricted expression in normal adult tissues, CTAs have been attractive targets for biomarker development [11] and could serve as unique biomarkers for cancer diagnosis and prognosis [12–15]. CTAs are widely studied in tumors of different histotypes and although some studies have shown that CTA genes are not universally expressed in all tumour types [16–18], based on RT-PCR analysis it has been indicated that various members of CTAs are expressed in different types of cancers such as melanoma, lymphoma, bladder, breast, prostate, kidney, colon, and nonsmall cell lung cancers [19–22].

A variety of studies have reported the expression of different members of CTA genes in CRC [9, 23–25]. The actual frequency of CTA genes expression varies substantially between different studies performed on CRC and we cannot ignore the lack of uniformity in analytical techniques as a source of data variation. Recently, some investigations have been carried out using RT-PCR and statistical analysis to reveal the correlation of some CTA genes expression frequency and clinical risk factors with malignancy in CRCs [8, 26]. In the current study, implementing the same molecular technique and statistical analysis on our population, we aimed to confirm the impact of three CTA genes expression frequency and some clinical risk factors in both primary tumors with CLM and primary tumors with no CLM in patients with CRC. The selection of these three CTA genes was based on two key reasons: (1) their presence in colorectal cancer tissue or cell lines and (2) their association with tumour aggressiveness [8, 26].

## 2. Materials and Methods

### 2.1. Patients

Ninety patients with locally advanced colorectal cancer admitted to Rasol Akram Hospital in Tehran were enrolled in this study. The project was approved by the local ethics committee of Rasol Akram Hospital and written informed consent was obtained from each case. The colorectal cancer patients had received surgical but not any chemotherapy treatment. During the follow-up period between 2008 and 2013, liver metastases were observed in 36 (47%) cases. Clinicopathological characteristics contained demographic variables (age, gender), tumor size, tumor location (colon and rectum), and pathological status classified according to the TNM system [15]. Fresh tissue specimens including primary tumors with no CLM, primary tumors with CLM tissue, and paired adjacent normal tissue were collected by the clinicians in separated sterile tubes. Tissue samples were frozen and stored at −70°C.

### 2.2. RNA Extraction and cDNA Synthesis

Total RNA extraction was performed from 50–100 mg of each sample with the TriPure Isolation Reagent (Roche Applied Sciences, Germany). For cDNA synthesis, 3 *μ*g of total RNA from each sample was used to synthesize first-strand cDNA according to the manufacturer protocol (Fermentas, Germany).

### 2.3. Reverse Transcriptase-Polymerase Chain Reaction

To evaluate the expression of individual CTA genes, all reactions were carried out in a peqSTAR 96 Universal Thermal Cycler. The PCR mixture included 1 *μ*M primer, 200 *μ*M of each dNTP (KBC), reaction buffer 1x with 1.5 mM mgCl_2_, and 1 unit Taq polymerase (5 U/*μ*L, KBC). The PCR analysis was performed on selected genes using the primers and the conditions shown in Table 1. Amplified segments were analyzed by electrophoresis on a 2.5% agarose gel, stained with ethidium bromide, and observed under E box vx2 ultraviolet light system.

### 2.4. Statistical Analysis

All the data were tabulated, and the statistical analysis was performed using the SPSS software V16.0 (SPSS, Inc., Chicago, IL). Association of the frequency of CTA genes expression and clinicopathological markers with CLM was analyzed using Fisher's exact test for a two-by-two contingency table or by the Pearson *χ* ^2^ test. The multiple logistic regression was performed to determine those independent factors which were significantly important in predicting CLM.

In all statistical analyses, a *P* value less than 0.05 was considered to be statistically significant. Finally, the coexpression of the CTA genes as well as the association of CTA genes coexpression with CLM was analyzed using Fisher's exact test. All of the variables assessed in this study were categorical except for “age” and “tumour size” and hence to create the two-by-two contingency table, we divided each of them into two groups according to their calculated means (Table 2).

## 3. Results

### 3.1. Clinical Samples

Samples used in this study including primary tumors without CLM, primary tumors with CLM, and adjacent normal tissues were collected from 90 CRC patients. However, clinicopathological data was available only for 77 patients including 37 (48%) men and 40 (52%) women, ranging in age from 17 to 80 years (median 51 years). Table 2 represents the information related to patients' clinicopathological markers.

### 3.2. Results of RT-PCR Amplification

**Figure 1:**
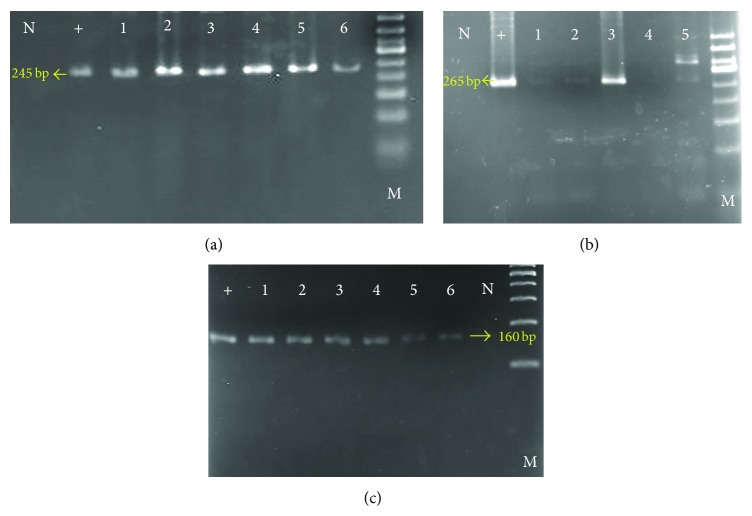
The figures show the result of RT-PCR analysis of positive mRNA expression of CTA genes in some patients. Each band represents a positive mRNA expression of CTA genes: (a), (b), and (c):* PAGE4*,* SCP-1*, and* SPANXA/D*, respectively. M: molecular marker; N: negative control; +: positive control.

CTA mRNA expression was examined with RT-PCR in primary tumors, liver metastasis, and paired adjacent normal tissues in each CRC patient. None of these genes were expressed in normal mucosal tissues.  Figure 1 illustrates positive RT-PCR results for each CTA gene studied.

### 3.3. Correlation between CTA mRNA Expression and CLM

The results of Pearson *χ* ^2^ test and fisher exact test showed higher frequency of *PAGE4 * and* SCP-1* genes expression in primary tumors with CLM than that in primary tumors without CLM and the difference was statistically significant (*P* < 0.05).* SPANXA/D* gene expression was also detected at higher frequencies in primary tumors with CLM, but the difference with primary tumors without CLM was not statistically significant. Nevertheless, there was no significant difference in expression patterns of CTA genes between these two categories of CRC (Table 3).

### 3.4. Correlation between CTA mRNA Expression and Clinicopathological Markers

The association between clinical risk factors and the expression frequency of three CTA genes were examined (Table 3). Our results revealed that the expression of* SCP-1* and* PAGE4* was significantly correlated with lymph node metastasis (N category) (*P* < 0.05).

### 3.5. Clinicopathological Parameters in Patients with CLM

We evaluated the correlation between clinical risk factors and primary tumours with CLM. The statistical results suggested that the depth of invasion (*P* = 0.011) and lymph node metastasis (*P* = 0.001) were associated with liver metastasis of CRC.

### 3.6. Coexpression of CTA Genes in CRC

Of all 90 CRC patients, 46 patients (51%) expressed at least one CT gene. Among them, 23 patients (50%) expressed one CT gene and the other 23 patients (50%) expressed two or three CT genes. Only the expression of* SCP-1* was significantly associated with* PAGE4* and* SPANXA/D* genes expression (*P* < 0.05) (Table 4).

### 3.7. Correlation between Coexpression of CTA Genes and CLM in Patients with CRC

In this study, 48% of patients with CRC showed CLM, among which 17% had no expression of three genes, 28% had coexpression of two genes, and 11% had coexpression of three genes (Table 5).

### 3.8. Binary Logistic Regression Analysis

Binary logistic regression analysis with maximum likelihood estimation was accomplished with 4 potential risk factors for liver metastasis (*SCP-1*,* PAGE4*, depth of invasion, and lymph node involvement). We found a statistically significant relationship between both* PAGE4* (*X*_1_) and lymph node involvement (N1: *X*_2_, N2: *X*_3_) with CLM (*P* < 0.05) (Table 6) and therefore they were included in the multiple logistic regression model for liver metastasis risk prediction.* PAGE4* and lymph node involvement were identified as independent risk factors for CLM (*P* < 0.05).

The following equation was used to predict estimated risk of liver metastasis in CRC patients:

(1) $$\begin{array}{c} Px=\frac{e^{\left(-2.497+2.627X_{1}+2.373X_{2}+2.249X_{3}\right)}}{1+e^{\left(-2.497+2.627X_{1}+2.373X_{2}+2.249X_{3}\right)}}. \end{array}$$

The classification accuracy rate of the model based on* PAGE4* and lymph node metastases was 80.3% which surpassed the proportional by chance accuracy criteria, supporting the utility of the model.  Table 7 shows predicted risk of liver metastases based on possible results of the model compared with actual observation of liver metastasis from CRC patients in this dataset.

In patients with none of the two* PAGE4* and lymph node metastases markers, the actual percentage of CRC patients with liver metastasis was 6.9%, whereas the predicted risk based on the model was 7.6%. When lymph node involvement, depending on the type of N category (*N*1, *N*2), was present, the actual percentage of CRC patients with liver metastasis ranged from 43% to 50%, and predicted risk ranged from 44% to 46%. When both risk factors existed, the actual proportion of CLM was 94%, and risk calculated by the model was 92%. Thus, adding* PAGE4* to the classical risk factor of lymph node involvement improved the predictive power for liver metastasis by nearly 40%.

## 4. Discussion

Colorectal cancer is the third common carcinoma with a high rate of mortality around the world, corresponding to the second cause of cancer-related death [27]. In most cases, death results from the formation of secondary neoplasms called metastases. Over the last decades, tremendous studies about cancer molecular markers have been accomplished; however, only a few such markers have entered clinical practice. The lack of clinical prognostic markers clearly reflects limitations in prognostic studies. On the other hand, between-study heterogeneity in evaluating the role of biomarkers in cancer necessitates confirmatory studies to validate primary studies.

To date, about 83 CTA gene families including more than 140 members have been discovered [28]. Although the function of CTA genes is still largely unknown partly due to their presence in multiple tumor types, their limited expression in normal tissue has made them ideal molecular markers for cancer prognosis and diagnosis [29, 30]. To improve the prognosis of CRC, the most significant considerations are the selection of patients at high risk for liver metastasis and subsequently the initiation of suitable adjuvant therapy. Adjuvant therapy in patients with CRC after curative resection has been reported to be useful for improving overall and disease-free survival [31, 32].

Although, some scientists initially hypothesized that there must be an obvious difference in gene expression profiles between primary tumours with no CLM and primary tumours with CLM (because they assumed that primary cancer cells must obtain the potential of metastasis during late tumorigenesis steps), in this study we found no apparent difference in CTA expression pattern between these two categories of CRC. This finding confirmed that CTA genes expression pattern is maintained during CRC liver metastasis. Our results are consistent with the studies of Chen et al. and Alves et al., who reported no difference in expression pattern of understudied CTA genes between primary tumours with no CLM and primary tumours with CLM [8, 9]. Moreover, these two studies have shown that there are similar expression profiles in the two CRC categories obtained from the same individuals. Also, another study on breast cancer has delineated that metastasis development in individuals is related to their different genetic backgrounds; in other words, metastasis may be unavoidable for patients with certain genetic profiles, while for others, it may never arise [33]. Our results together with these studies have thrown the initial hypothesis into question.

According to our findings, there is a statistically significant higher frequency in expression of* PAGE4* and* SCP-1* genes in primary tumours with CLM when comparing to primary tumors with no CLM (*P* < 0.05), suggesting that the expression of these two genes might have a correlation with the process of liver metastasis. This result confirms the study outcome of Chen et al. who reported that the expression frequency of* PAGE4* and* SCP-1* genes in colorectal cancer has significant correlation with liver metastasis [8]. Some studies on other types of cancer also revealed similar results for the association of* PAGE4* gene expression with aggressive phenotypes [34, 35]. In addition, a recent study has shown that silencing and overexpressing of PAG4 mRNA in prostate cancer xenografts are linked, respectively, to induction of cell death via apoptosis and protection of cells from stress-induced death [36]. The correlation between higher frequency of* SCP-1* gene expression and high graded tumor was also reported by Tammela et al. who worked on epithelial ovarian cancer (EOC) [37].

According to our statistical analysis, there was no significant difference of* SPANXA/D* gene expression frequency between primary tumours with CLM and primary tumours with no CLM. In contrast, Chen et al. reported that the frequency of SPANX gene expression was significantly higher in primary tumours with CLM than that in primary tumours with no CLM though they did not determine the subfamily of SPANX that they studied [8].

The association between CTA genes expression frequency and clinicopathological factors was also examined and our data showed that the expression of* PAGE4* and* SCP-1* was correlated with the presence of lymph node metastasis. Kong et al. in a study on* PAGE4* demonstrated that the expression frequency of this gene was correlated with lymph node metastasis in gastric cancer patients [34], while in a study on colorectal cancer, it has been reported that there was no significant correlation between* PAGE4* gene expression frequency and lymph node metastases [8]. In terms of* SCP-1* gene, Chen et al. demonstrated that there is a significant correlation between its expression frequency and the presence of lymph node metastasis in patients with CRC [8]. Therefore, our findings suggested that both* PAGE4* and* SCP-1* genes expression frequencies are associated with malignant phenotypes in CRC, especially during both local invasion and distant metastasis. However, the expression frequency of all three CTA genes was independent of other clinical factors (Table 1).

Of all clinicopathological factors investigated in this study, the frequency of lymph node metastasis and depth of tumour invasion were significantly correlated with CLM. This finding supports the result obtained from the study of Chen et al. [8].

Interestingly, some reports suggested that multiple CTA genes tend to be coexpressed in the same tumor. Li et al. by investigating coexpression of some CTA genes in 121 CRC patients revealed that 56.2% expressed at least one CT gene. Among them, 28.9% expressed one CT gene and 27.3% expressed more than two CT genes [14]. Our study on 90 CRC patients showed that 51% expressed at least one CT gene. Among them, 50% expressed one CT gene and 50% expressed two or three CT genes. We also assessed the correlation of these coexpressions between primary tumours with CLM and primary tumours with no CLM. The results showed that coexpression of CTA genes was higher in primary tumours with CLM than in primary tumours with no CLM and the difference was statistically significant (*P* < 0.001).

In the current confirmatory study, we established a formula to predict liver metastasis in patients with CRC. All factors significantly correlated with liver metastasis (the frequency of* PAGE4* and* SCP-1* genes expression, depth of invasion, and lymph node metastasis) were taken into account for performing multiple logistic regression analysis. However, only two independent factors—*PAGE4* and lymph node metastasis—were selected as candidate markers for establishing a panel to predict liver metastasis in CRC patients. The classification accuracy of the model was 80.3% and this panel could show high similarity between observed and predicted risk of liver metastasis (Table 7). Interestingly, adding* PAGE4* to the clinical risk factor of lymph node involvement could improve the power of liver metastasis prediction by approximately 40%. The regression model constructed by Chen et al. was based on three independent risk factors including* PAGE4*, lymph node metastasis, and vessel cancer embolus status. According to their report, involving PAGE4to the two classical risk factors (vessel cancer embolus and lymph node metastasis) improved the predictive power for liver metastasis only by nearly 20% [8].

In conclusion, we could confirm the significant association between the frequency of* PAGE4* and* SCP-1* genes expression and CRC liver metastasis; however, we did not verify this association for* SPANXA/D*. We also confirmed that the frequency of lymph node metastasis and depth of tumour invasion were significantly correlated with CRC liver metastasis. Although we verified the correlation between* SCP-1* and lymph node metastasis, the lack of correlation between* PAGE4* and lymph node metastasis was rejected in our dataset.

We believe that constructing a more accurate predictive model based on molecular markers and their subsequent validation can bring a new horizon in diagnosis/prognosis of colorectal cancer and the management of cancer patients.

## Figures and Tables

**Table 1:** Primers and condition of RT-PCR analysis.

Genes	Primers sequence (5′→3′)	Conditions			
		Denaturation	Annealing	Extension	Cycle (no.)
*PAGE4 *	GATGTGGTTGTATTCGTG ATCTCCTGCTTCTTTAGTC	94°C—1 min	57°C—30 s	72°C—1 min	35
*SCP-1 *	CCAAAGGCATATACAGTGAAGA CAGGGGTTGTTAGAGATGAAGG	94°C—1 min	62°C—45 s	72°C—1 min	35
*SPANXA/D *	GACAAACAATCCAGTGCC TCCTCCTGTAGCGAACCA	94°C—1 min	57°C—1 min	72°C—1 min	35

**Table 2:** Patients' clinicopathological data.

Risks factors	Tumour tissues	
	Negative liver metastases 53 (41)	Positive liver metastases 47 (36)
Gender		
Male (*n* = 37)	53 (22)	41 (15)
Female (*n* = 40)	47 (19)	59 (21)
Age		
>51 (*n* = 41)	53 (22)	52 (19)
<51 (*n* = 36)	47 (19)	48 (17)
Tumor size		
<5 (*n* = 40)	44 (18)	61 (22)
≥5 (*n* = 36)	66 (22)	39 (14)
Tumor location		
Colon (32)	39 (16)	45 (16)
Rectum (45)	61 (25)	55 (20)
Depth of tumor invasion∗ (T factor)		
T1 (*n* = 0)	0	0
T2 (*n* = 25)	49 (20)	14 (5)
T3 (*n* = 45)	46 (19)	72 (26)
T4 (*n* = 7)	5 (2)	14 (5)
Lymph node metastasis∗ (N factor)		
N0 (*n* = 38)	76 (31)	19 (7)
N1 (*n* = 25)	12 (5)	56 (20)
N2 (*n* = 14)	12 (5)	25 (9)

Data are percentage of patients with number in parentheses.

^*^P < 0.05 for the comparison primary tumors without CLM versus primary tumors with CLM.

**Table 3:** Correlation between clinicopathologic risk factors and expression frequency of CTA.

Risk factors	*PAGE4 *			*SCP-1 *			*SPANXA1 *		
	−	+	*P* value	−	+	*P* value	−	+	*P* value
Gender									
Male (*n* = 37)	59	41	0.818	76	24	0.617	16	84	1
Female (*n* = 40)	55	45		70	30		15	85	
Age									
>51 (*n* = 41)	54	46	0.645	71	29	0.799	80	20	0.645
<51 (*n* = 36)	61	39		75	25		89	11	
Tumor location									
Colon (32)	40	60	0.493	49	51	0.262	30	70	0.365
Rectum (45)	37	63		60	40		38	62	
T category									
T1 (*n* = 0)	0	0	0.057	0	0	0.143	0	0	0.482
T2 (*n* = 24)	75	25		79	21		83	17	
T3 (*n* = 45)	53	47		76	24		82	18	
T4 (*n* = 7)	29	71		43	57		100	0	
M category									
M0 (*n* = 41)	85	15	0.001∗	85	15	0.011∗	85	15	1
M1 (*n* = 36)	25	75		62	38		83	17	
N category									
N0 (*n* = 38)	76	24	0.002∗	87	13	0.003∗	92	8	0.181
N1 (*n* = 25)	28	72		48	52		76	24	
N2 (*n* = 14)	57	43		79	21		79	21	
Tumor size									
<5 (*n* = 38)	53	47	0.355	74	26	0.798	80	20	0.623
≥5 (*n* = 36)	64	36		69	31		67	33	

Data are percentages of patients with (+) or without (−) the gene expression.

∗Statistically significant, *P* < 0.05.

**Table 4:** Association between the expressions of three CTA genes.

CTA genes	*SCP-1 *		*P* value	*SPANAXA/D *		*P* value
	+	−		+	−	
*PAGE4 *						
+	45	55	0.004∗	21	79	0.342
−	14	86		11	89	
*SCP-1 *						
+				48	52	0.000∗
−				4	96	

Data are percentages of patients with (+) or without (−) the gene expression.

∗Statistically significant, *P* < 0.05.

**Table 5:** Association of coexpression of 3 cancer/testis antigens with liver metastasis.

Tumour tissues	Expression statute				
	No expression	One CTA expression	Two CTA expressions	Three CTA expressions	*P* value
Negative liver metastases CRC (M0)	74	17	2	7	0.000∗
Positive liver metastases CRC (M1)	17	44	28	11	

Data are percentages of patients.

∗Statistically significant, *P* < 0.05.

**Table 6:** Binary logistic regression analysis of expression of *PAGE4* (*X*_1_) and lymph node involvement (N1, N2).

	Regression coefficient	SE	Odd ratio	95% Confidence interval		*P* value
*PAGE4 * (*X*_1_)	2.627	0.682	13.833	3.634	52.650	0.000∗
N1 (*X*_2_)	2.373	0.762	10.735	2.409	47.842	0.002∗
N2 (*X*_3_)	2.249	0.834	9.483	1.849	48.635	0.007∗
Constant	−2.497	0.601	0.082			

SE: standard error.

∗Statistically significant, *P* < 0.05.

**Table 7:** Possible combinations of *PAGE4* and lymph node involvement (N1, N2) for assessing the utility of the model.

Possible combination	PAGE4	N2	N1	Number of patients	Actual risk %	Predicted risk %
1	−	−	−	29	6.9	7.6
2	−	−	+	7	43	46
3	−	+	−	8	50	44
4	+	−	−	9	56	53
5	+	+	−	6	83	92
6	+	−	+	17	94	92
